# Identifying biomarkers of hepatic fatty acid metabolism disorder in sevoflurane-induced brain developmental injury by bioinformatics analysis

**DOI:** 10.3389/fnmol.2025.1369365

**Published:** 2025-09-04

**Authors:** Haozheng Yuan, Yuying Lv, Pei Fan, Pengyu Jia, Kui Wang, Kailing Hu, Haodong Sun, Xinlin Chen, Pengbo Zhang

**Affiliations:** ^1^Department of Anesthesiology, The Second Affiliated Hospital of Xi’an Jiaotong University, Xi’an, Shaanxi, China; ^2^Institute of Neurobiology, The Medical College of Xi’an Jiaotong University, Xi’an, Shaanxi, China

**Keywords:** sevoflurane, neurodevelopmental toxicity, hepatic fatty acid metabolism, immune cell infiltration, bioinformatics analysis

## Abstract

**Introduction:**

Exposure to sevoflurane in neonatal rats disrupts energy metabolism during brain development, which is associated with anesthetic-induced neurodevelopmental toxicity. Hepatic fatty acid metabolism plays a critical role in response to brain energy supply. However, how sevoflurane exposure affect hepatic fatty acid metabolism remains unclear.

**Methods:**

We employed multiple analytical methods in neonatal rats following sevoflurane exposure to: (1) Analyze alterations in hepatic fatty acid metabolism-related gene expression and immune cell infiltration; (2) Decipher associated metabolic pathways, including cholesterol metabolism and the expression changes of Dhcr24; (3) Conduct enrichment analyses (GO, KEGG, GSEA, GSVA) and functional investigations via Friends analysis; (4) Construct mRNA-miRNA-lncRNA regulatory networks; (5) Identify key genedrug small molecule interactions based on IC50 differences and (6) Verify the expression of key genes involved in fatty acid metabolism and the activation of immune cells.

**Results:**

Significant alterations were observed: (1) Identification of 15 key fatty acid metabolism-related differentially expressed genes (DEGs and RT-PCR); (2) Significant enrichment of 40 GO terms and 5 KEGG pathways; (3) GSEA/GSVA revealed 130 up-regulated and 62 down-regulated GO gene sets, along with 5 up-regulated and 2 down-regulated KEGG pathways; (4) Friends analysis highlighted Dhcr24 as a critical player in cholesterol metabolism; (5) Network analysis identified pivotal mRNA and lncRNA nodes within the regulatory networks; (6) Screening yielded 43 key gene-drug combinations with significant IC50 differences; and (7) Immunofluorescence confirmed the activation expression of relevant immune cells. Bioinformatics analysis pinpointed diagnostic biomarkers for both hepatic fatty acid metabolism perturbations and immune cell infiltration following exposure.

**Discussion:**

These findings demonstrate that neonatal sevoflurane exposure profoundly affects hepatic fatty acid metabolism and immune cell infiltration, involving specific key genes (including Dhcr24), perturbed pathways, and regulatory networks. The identified biomarkers and potential therapeutic targets provide a crucial foundation for developing more specific countermeasures against sevoflurane-induced neurodevelopmental toxicity, potentially via targeting the liver-brain metabolic axis.

## 1 Introduction

Sevoflurane is an inhaled general anesthetics that is widely used for induction and maintenance of general anesthesia across all age groups and surgical sites. It was shown that exposure to sevoflurane at a critical stage of brain development impaired learning and memory (Apai et al., 2021; Qiu et al., 2024). The underling mechanism has not been fully understood. Several studies (Liu et al., 2015; Ju et al., 2017; Yang et al., 2021) have confirmed that neonatal sevoflurane anesthesia inhibits the metabolism of fatty acids in liver and brain tissue in neonates, causing systemic energy deficiency and neuron injury. However, further research is required to reveal that how sevoflurane exposure affect hepatic fatty acid metabolism and whether there are diagnostic biomarkers for fatty acid metabolism in neonates.

The liver is a vital organ that utilizes fatty acids to generate energy through a multi-step process, which involves beta-oxidation and beta-hydroxybutyric acid production (Molenaar et al., 2017). The developing brain relies on glucose and ketone bodies produced by the liver cells as energy substrates (Antunes et al., 2024). In addition to the metabolic functions, the liver is an important immune organ. It was shown that a well-regulated immune system is needed for proper brain development and function (Filiano et al., 2015). Furthermore, the liver and brain have their unique dialogue to transmit metabolic messages (Yang et al., 2024). Thus, we proposed that impairment of learning and memory following sevoflurane exposure was the consequence that sevoflurane disturbed liver metabolism and immunity of neonatal rats. Lymphocytes, including helper/induced T lymphocytes (CD3 + CD4+) and inhibitory/cytotoxic T lymphocytes (CD3 + CD8+), are a crucial cell population in the immune system. The CD4+/CD8+ ratio reflects change of immune system, and a decreased ratio often indicates a serious illness and poor prognosis (Hadj-Moussa et al., 2021). B cells play a crucial role in specific humoral immunity through producing antibodies. They also act as important antigen-presenting cells. NK cells, on the other hand, are crucial immunoregulatory cells that can regulate T cells, B cells, and bone marrow stem cells. They control the immune function by releasing lymphokines (Wang et al., 2014). It was reported that exposure to sevoflurane reduced the peripheral blood lymphocyte (Elena et al., 2003), indicating that sevoflurane exposure affected immunity. However, the change of immune cell in liver after sevoflurane exposure remains unclear.

**Figure 1:**
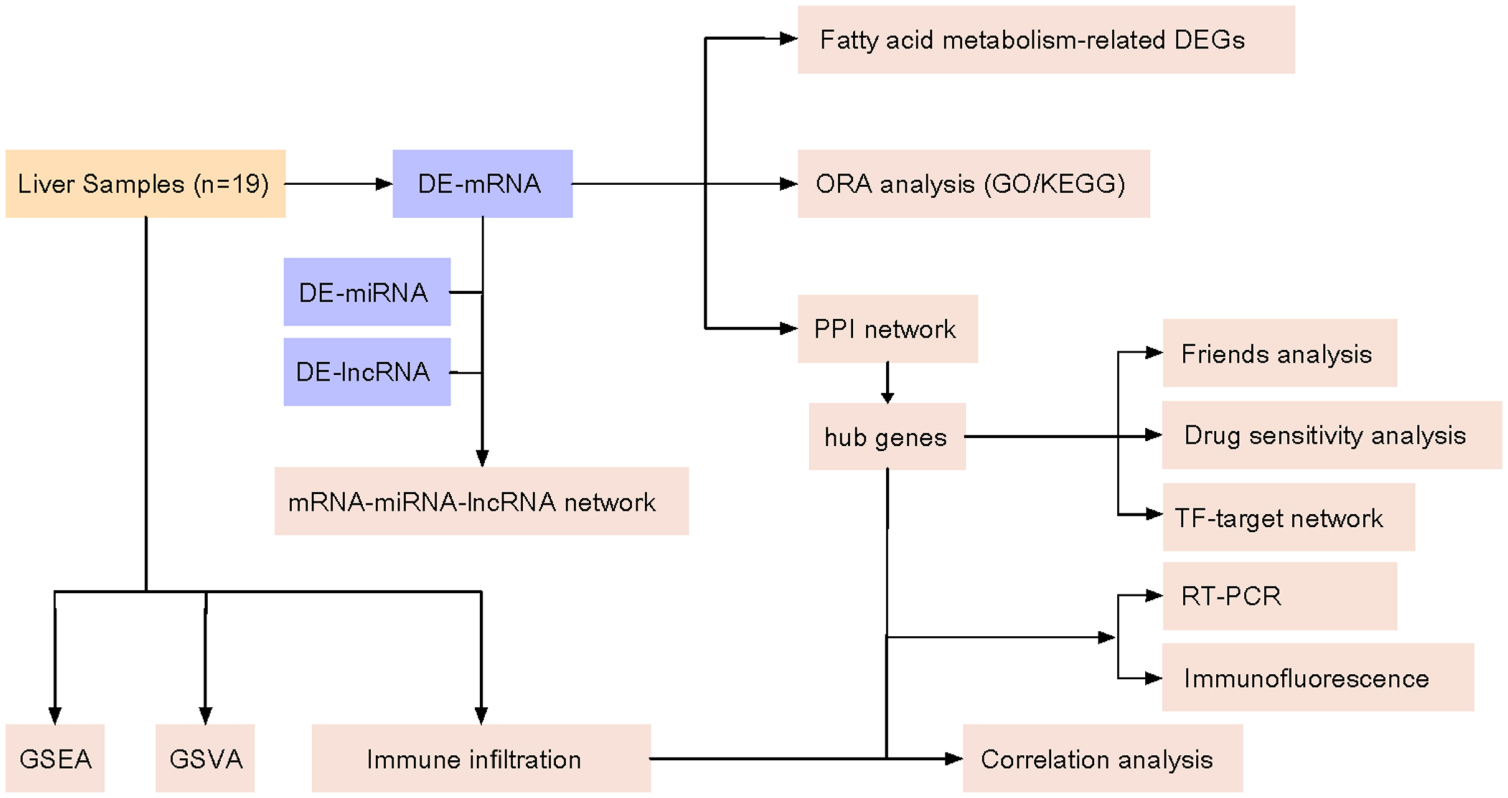
Technology roadmap. Differential gene expression (DEG); gene ontology (GO); Kyoto Encyclopedia of Genes and Genomes (KEGG); messenger RNA (mRNA); protein–protein interaction (PPI); microRNA (miRNA); long noncoding RNA (IncRNA); gene set enrichment analysis (GSEA); gene set variation analysis (GSVA); transcription factor (TF).

In this study, we used high-throughput sequencing technology to perform whole-genome transcriptome sequencing on sevoflurane-exposed neonatal rat liver to obtain a large amount of gene expression data. Subsequently, bioinformatics methods were used to process and analyze these data, screen out genes closely related to fatty acid metabolism, and further to verify their expression changes in sevoflurane-exposed neonatal rats. Through in-depth study of potential biomarkers, we will understand the mechanism of that sevoflurane influences fatty acid metabolism in rat liver. In addition, the biomarkers may be a powerful tool for assessing neurodevelopmental toxicity of sevoflurane and predicting an individual sensitivity to it, which will provide an important theoretical basis for the safety evaluation of sevoflurane in neonatal applications. The technical route of this study is shown in Figure 1. After transcriptome sequencing of 19 rat liver samples, the regulatory mechanism of sevoflurane exposure on fatty acid metabolism was systematically explored through differential analysis, multi-omics integration, and functional verification.

## 2 Materials and methods

### 2.1 Animal model and grouping

The experimental protocol was approved by the Xi’an Jiaotong University Laboratory Animal Administration Committee (No. 2020-4). Calculated from sample size, 19 postnatal day (PND) 7 rats (*Rattus Norvegicus*), weighing 15–18 g with males and females, from the Laboratory Animal Centre of Xi’an Jiaotong University were divided randomly into control group (*n* = 8) and sevoflurane group (*n* = 11), To minimize experimental error, the rats in the two groups were subdivided control (C1–C3) and (C4–C8), as well as sevoflurane (S1–S3) and (S4–S11), respectively. According to previous report, the rats in sevoflurane group were placed in a placed into an R-CU500-PRO small animal ICU incubator (RWD corporation) to maintain body temperature constant at 37°C, loaded with 60% air-oxygen mixture, then exposed to 3.4% sevofurane (Hengrui Pharmaceutical Group Corporation, Shanghai, China) in a 2 L min^−1^ gas mixture for 6 h. The gas mixture entered from the upper air inlet on the left side of the anesthetic chamber through a threaded tube and then discharged from the lower air outlet on the right side of the anesthetic chamber. The exhaust gas was connected to a gas analyzer (Drager Inc., German) to monitor the concentrations of sevofurane and CO_2_. The pulse oxygen saturation (SpO2) was measured on the abdomen of anesthetized pups using an infant pulse oximetry probe. The rats in control group were placed in a holding box with the same concentration of oxygen for 6 h. The absence of hypoxia and carbon dioxide retention was also verified by testing arterial blood gas analysis. At the end of the exposure, 300–500 mg of liver was retained by ice saline infusion and then quickly frozen in liquid nitrogen for 3 min before being transferred to −80 degrees Celsius refrigerator for freezing and preservation.

### 2.2 Data acquisition and pre-processing

19 PND7 rats liver samples were used to sequence the whole transcriptome (mRNA, lncRNA and miRNA) based on a DNA sequencing (DNA-seq) platform. A total of 11 samples were suppressed after sevoflurane exposure and the other 8 samples were used as controls. The above sequencing data were quality-controlled and then analyzed the downstream to obtain the expression matrix. In order to ensure the accuracy and reliability of subsequent analysis, the following measurements were adopted. (1) Quality control: the data quality of all samples was first assessed using the FastQC software to check for low quality areas or contamination. For unqualified data, the problem sequence was resequenced or removed from the sample. (2) Removal of adapter sequences: since additional artificial sequences (e.g., primers, adapters, etc.) may be introduced during library construction, the Trimmomatic tool is required to identify and remove these non-target fragments. (3) Filter low-quality reads: set a threshold criterion, such as bases with a Phred quality score below 20 will be marked as uncertain and removed from the data set. In addition, reads that are too short (usually less than 50 BP) were filtered out. (4) Align the reference genome: map the cleaned high-quality reads to the known rat reference genome, and use the STAR algorithm to complete this task. The purpose of this measurements is to determine the specific location information of each read and its corresponding gene annotation. (5) Quantification of expression: the relative abundance of each gene/transcript was calculated based on the alignment results. The commonly used methods are statistical models such as RSEM (RNA-Seq by Expectation Maximization) or DESeq2. (6) Outlier detection and handling: use the box plot or other visual means to identify the sample points that significantly deviate from the population distribution as potential outliers; further confirm their significance level through t test or ANOVA test to determine whether to retain them in the final data set for subsequent analysis. (7) Standardized adjustment: in order to eliminate the influence of technical variation that may exist between different batches, it is also necessary to compare the total readings of each sample and normalize them to make them comparable. Differential analysis was performed based on the DESeq2 package (Chen et al., 2022), with the threshold of |logFC| ≥ 1 and P. adjust ≤ 0.05 to obtain differential mRNA, lncRNA and miRNA.

### 2.3 Analysis of differential gene expression related to fatty acid metabolism

The keyword “fatty acid metabolism” was used to obtain h.all.v7.5.1.symbols.gmt, c2.cp.kegg.v7.5.1.symbols.gmt and c2.cp.reactome.v7.5.1.symbols.gmt through Molecular Signature Database (MSigDB, v7.5.1) package (Chen et al., 2021). kegg.v7.5.1.symbols.gmt and c2.cp.reactome.v7.5.1.symbols.gmt for fatty acid metabolism-related datasets (Rollins et al., 2019; Yu et al., 2022). After removing duplicate genes from different datasets, 299 fatty acid metabolism-related genes were obtained (Table 1). The fatty acid metabolism-related genes were jointly taken as intersection with differentially expressed genes and visualized by ggheatmap for heat map based on their expression levels (Liu et al., 2022).

**Table 1:** GO ORA partial enrichment results.

Description	Ontology	*p* value	p.adjust	*q* value
Cholesterol biosynthetic process	BP	1.09E-07	1.05E-05	6.43E-06
Sterol biosynthetic process	BP	1.45E-07	1.05E-05	6.43E-06
Steroid biosynthetic process	BP	1.70E-07	1.05E-05	6.43E-06
Cholesterol metabolic process	BP	4.01E-06	0.000165521	0.000101167
Sterol metabolic process	BP	4.89E-06	0.000165521	0.000101167
Fatty acid metabolic process	BP	1.74E-05	0.000448942	0.000274396
Peroxisome	CC	9.42E-08	1.13E-06	8.43E-07
Microbody	CC	9.42E-08	1.13E-06	8.43E-07
Oxidoreductase activity, acting on the CH-OH group of donors, NAD or NADP as acceptor	MF	0.000220857	0.005602281	0.002307576
Oxidoreductase activity, acting on CH-OH group of donors	MF	0.000278175	0.005602281	0.002307576
NAD binding	MF	0.001867062	0.021471214	0.008843978

BP, biological process; CC, cellular component; MF, molecular function.

### 2.4 Functional and pathway enrichment analysis of differentially expressed genes

Gene Ontology (GO) and the Kyoto Encyclopedia of Genes and Genomes (KEGG) (Zhao et al., 2020) are common databases for enrichment studies. GO contains three sections: biological process (BP), molecular function (MF) and cellular component (CC), which could systematically annotate the properties of genes and their products. The KEGG focuses on genomes, enzymatic pathways and biochemicals, and can be used to query the network of molecular interactions in cells. We performed enrichment analysis of differential expressed genes based on the org.Rn.eg.db (3.15.0) database using the clusterProfiler package (Liu et al., 2022), correcting the *p*-value by the Benjamini-Hochberg (BH) method, with a threshold of corrected P.adjust less than or equal to 0.05. The significantly enriched gene set was obtained. Visualization was performed using ggplot2 (Gustavsson et al., 2022), enrichplot (Li et al., 2022) and Cytoscape (Zhang et al., 2022).

### 2.5 Gene set enrichment analysis

Gene Set Enrichment Analysis (GSEA) is used to evaluate the trend of distribution of genes in a predefined gene set in a table of genes ordered by their phenotypic correlation and thus determine their contribution to the phenotype (Yang et al., 2023). In this study, GSEA of samples was performed using the clusterProfiler package based on the c5.go.v7.5.1.symbols.gmt and c2.cp.kegg.v7.5.1.symbols.gmt gene set. This enrichment analysis was performed using the following parameters: each gene set contained a minimum of 10 genes and a maximum of 500 genes, and the *p*-value correction method was Benjamini-Hochberg (BH). Significantly different gene sets were obtained with a P.adjust less than or equal to 0.05 as the threshold and visualized by the enrichplot package.

### 2.6 GSVA enrichment analysis

Gene set variation analysis (GSVA), is a non-parametric unsupervised analysis method that is used to evaluate the gene set enrichment results of the microarray nuclear transcriptome by converting the expression matrix of genes between samples into the expression matrix of gene sets between samples. Thus, to assess whether different pathways are enriched between different samples (Shi et al., 2022). In this study, GSVA was performed on rat liver samples by GSVA package based on c5.go.v7.5.1.symbols.gmt and c2.cp.kegg.v7.5.1.symbols.gmt gene sets obtained from MsigDB database, and differential analysis of enrichment results was performed using limma package (Shi et al., 2022), with logFC absolute values greater than or equal to 0.5 and P.adjust less than or equal to 0.05 as the threshold to obtain significantly different gene sets.

### 2.7 Analysis of protein–protein interaction networks

Protein–Protein Interaction (PPI) Networks are composed of proteins that interact with each other and are closely related to various aspects of life processes such as biological signaling and regulation of gene expression. STRING database can be used to retrieve known or predicted protein interactions (Xie et al., 2022). In this study, the screened differential genes were imported into the STRING database, and the appropriate query parameters were set (confidence was set to 0.7), so that those interactions considered to have high confidence were included in the network. This selection criterion helps to filter out unreliable data, thus enhancing the effectiveness and accuracy of network construction. After obtaining the protein–protein interaction data returned from the STRING database, we imported these data into Cytoscape software for visual analysis. Cytoscape is a powerful open-source software platform that can be used to map complex networks and help researchers intuitively understand the interactions between proteins. With the corresponding modular setup, the resulting PPI network will contain nodes (representing different proteins). And edges, which represent interactions between them, with the size of the nodes adjusted according to the number or strength of interactions. Once the network was constructed, we utilized Cytoscape’s cytoHubba plugin for further analysis. In this study, we adopt the MCC (Maximum Clique Centrality) algorithm to evaluate the importance of each gene in the network (Zeng et al., 2022). The MCC algorithm identifies important genes that play a key role in the network structure by considering the connectivity and interaction patterns of nodes. Based on the calculation results of MCC algorithm, we identified the first six differential genes as hub genes. To further visualize the distribution of these key genes in the genome, we used the RCircos tool. RCircos is a visualization tool that combines genomic data with interaction networks to generate circular graphs that integrate genomic location and interaction information. In RCircos, we annotate the chromosomal location information of key genes, so that we can quickly identify the specific location of key genes on the genome and their clustering on specific chromosomes (An et al., 2015).

### 2.8 Key gene friends analysis

The semantic comparison of GO annotations provides a quantitative method to calculate the similarity between genes and genomes. In this study, the semantic similarity of key genes was derived based on the GOSemSim package (Bi et al., 2021), and the final score was obtained by calculating their geometric mean at different levels of GO (BP, MF and CC) and visualized using ggplot2.

### 2.9 Differential mRNA-differential miRNA-differential lncRNA network analysis

Competing endogenous RNA (ceRNA) represents a novel mode of gene expression regulation, which could compete for miRNA binding and deregulate miRNAs to enhance target gene expression. mRNAs and lncRNAs could “communicate” with each other through MREs (microRNA Response Elements), thus forming a genome-wide regulatory network. We have used miRanda (Zhu et al., 2022) and TargetScan (Li et al., 2023) to predict miRNA target genes and construct mRNA-miRNA-lncRNA networks for differential expressed genes related to fatty acid metabolism and visualize them by Cytoscape.

### 2.10 Transcription factor network construction for key genes

Transcription factor (TF) could interact with cis-acting elements of eukaryotic genes to activate or repress the transcription of genes. In this study, a transcription factor network was constructed for key genes by Network Analyst (Yue et al., 2022) based on the ENCODE (Yang et al., 2020) transcription factor-gene interaction database and visualized by Cytoscape.

### 2.11 Small molecule analysis of key gene drugs

To investigate the association between key genes and drug small molecule sensitivity, we then calculated the Pearson correlation coefficient between key gene expression levels and different drugs by using the CellMiner database (Zhou et al., 2022), and the results of the analysis were screened based on a *p* < 0.01 cut-off. The expression data were divided into two groups, high and low expression, separated by the median expression level of key genes, and the comparison between the two groups was performed based on a Wilcoxon rank sum test, and the gene-drug combinations with *p* < 0.01 were selected to draw box line plots based on ggpubr (Cheng et al., 2021).

### 2.12 Immune infiltration analysis

The immune microenvironment is an integrated system consisting mainly of immune cells and various cytokines and chemokines, etc., and immune infiltration analysis has an important guiding role in disease research, etc. CIBERSORT is an algorithm based on the principle of linear support vector regression for inverse convolution analysis, which can be used for estimation of cell type abundance (Zhou et al., 2022). To investigate the differences in immune infiltration between the two groups of samples, this study assessed the level of immune cell infiltration in each sample by IOBR package (Cheng et al., 2021) using the CIBERSORT algorithm based on the marker genes of 25 immune cells obtained from the literature, and then used ggplot2 to draw box plots and immune cell composition stacked histograms by ggstatsplot (He et al., 2022) to calculate the correlation between key genes and immune cells, and corrplot (Bi et al., 2022) to visualize the correlation between immune cells.

### 2.13 Liver gene expression

To verify the expression of different genes in the liver, we determined the expression levels of hepatic fatty acid metabolism-related genes Acot2, Dhcr24, Idi 1, Odc 1, Slc22a5, Acot12, Hsd17b7, Prkag2, Amcar, Mapkap1, HCCS, Idh and Elovl2 using real-time PCR. Briefly, RNA was extracted from the livers of neonatal rats after sevoflurane exposure using Trizol reagent (Invitrogen), and then reverse transcribed into cDNA using the PrimeScript™RT kit (Takara). cDNA levels of the target genes were amplified and quantified using the Bio-Rad iCycler Real-time PCR system. The cDNA level of the target gene was amplified and quantified using the Bio-Rad iCycler Real-time PCR System. The 2^−ΔΔCt^ method was used to calculate the gene expression level of each sample, which was repeated three times. The primer sequences are shown in Table 2.

**Table 2:** Primer sequences used for qRT-PCR.

Gene	Forward sequence	Reverse sequence
Acat2	AGAGAAACATACCCCAGGACAC	ATGAAGCAGGCATAGAGCAAAC
Dhcr24	ATGCTGGTACCCATGAAATGC	CGAGATCTTGTCATACACCTC
Idi1	ACAGGTTCAGCTTCTAGCAGA	CCTTTAAGCGCTTCTGTGCTG
Odc1	TGCTTGACATTGGTGGTG	TTCTCATCTGGCTTGGGT
Slc22a5	TGGGCAAGTTTGGAATCACC	CACCAAAGCTCTCTGGGAAG
Acot12	CATGGCGTGGATGGAGACA	TTGACGATGGCATTGAAGACAAG
Prkag2	CAGCGTCTCCTCCTCTCCATC	GCGGCTTTCTAGGCGTTCAG
Amcar	GACTCTACAGCAGGACAATCCAAAG	GGACACCTGACAAAGCCACATAG
Ugdh	ACATTAGCAAATACCTGATGGACGAG	CCGCTGAGACGCCTGGATG
Them5	CGTGGGCTCTCTGGCTGTC	CACTGTCTGCTTGTCTCTGCTC
Idh	ATGGCGGTTCTGTGGTGGAAATG	GGTCATTGGTGGCATCCCGATTC
Mapkap	ACCTGCTCTGTGCCTGTGAC	CGGTAGCTGCGTCGGACTC
Hccs	GATTGATTACTATGATGGCGGTGAG	AGCAACTTTCATTCTGTCCCATACTG
Elovl2	GCTTCTTCGGACCAACCCTGAAC	GCATGGACGGGAACACAGACAG

### 2.14 Immunofluorescence staining of liver tissue

Rats were deeply anesthetized with sodium pentobarbital (50 mg/kg) and then perfused transcardially with saline (4°C). Livers were fixed and then serially sliced into 10-μm-thick transverse sections using a cryosectioning machine (Leica, CM1950, Germany) and mounted on gelatin-coated glass slides. All slides were blocked with 10% goat serum for 2 h at room temperature and then incubated with the corresponding primary antibody overnight. Primary antibodies used were Mouse Anti-CD4 Antibody (1:200, YM3070, Immunoway), MouseNAnti-CD86 Antibody (1:200, ab238468, abcam), Rabbit Anti-Perforin 1 Antibody (1. 200, YT5792, Immunoway). The next day, the sections were rewarmed for 15 min, rinsed with PBS (3 times for 15 min each), and then incubated with the corresponding secondary antibodies for 2 h at room temperature. The secondary antibodies were CoraLite594 -conjucated Goat anti-Mouse IgG (1:200, SA00013-3, proteintech), CoraLite594 -conjucated Goat anti-Rabbit IgG (1:200, SA00013-4, proteintech), and CoraLite594 -conjucated Goat anti-Rabbit IgG (1:200, SA00013-4, proteintech), respectively. Sections were rinsed with PBS after DAPI re-staining (3 times for 15 min each). Fluorescence images were acquired using a microscope (Axioscope5, ZEISS, Germany). Four sections were taken from each liver, and 5 fields of view were taken from each section for cell counting. The average of 5 fields of view represented the number of positive cells in that section. The average of positive cells in 4 sections represented the average for that liver.

### 2.15 Statistical analysis

All analyzes in this article were based on R software (Version 4.2.0) and SPSS 13.0. Wilcoxon rank sum test was used to compare the differences between the two groups. Comparison of data between the two groups was done using *t* test. Pearson correlation coefficient was used to analyze the relationship between gene expression and drug sensitivity. This study involved multiple comparisons (e.g., comparison of expression levels of multiple genes, analysis of sensitivity to multiple drugs, etc.), which may increase the risk of false positive results. Therefore, in the statistical analysis, we used the Benjamini-Hochberg correction method to reduce this risk. By adjusting the *p*-value, it can control the increase of the first type error rate caused by multiple tests to a certain extent, so as to make the final conclusion more reliable. The results were based on *p* < 0.05 as the criterion for significantly different results.

## 3 Results

### 3.1 Differential gene analysis

**Figure 2:**
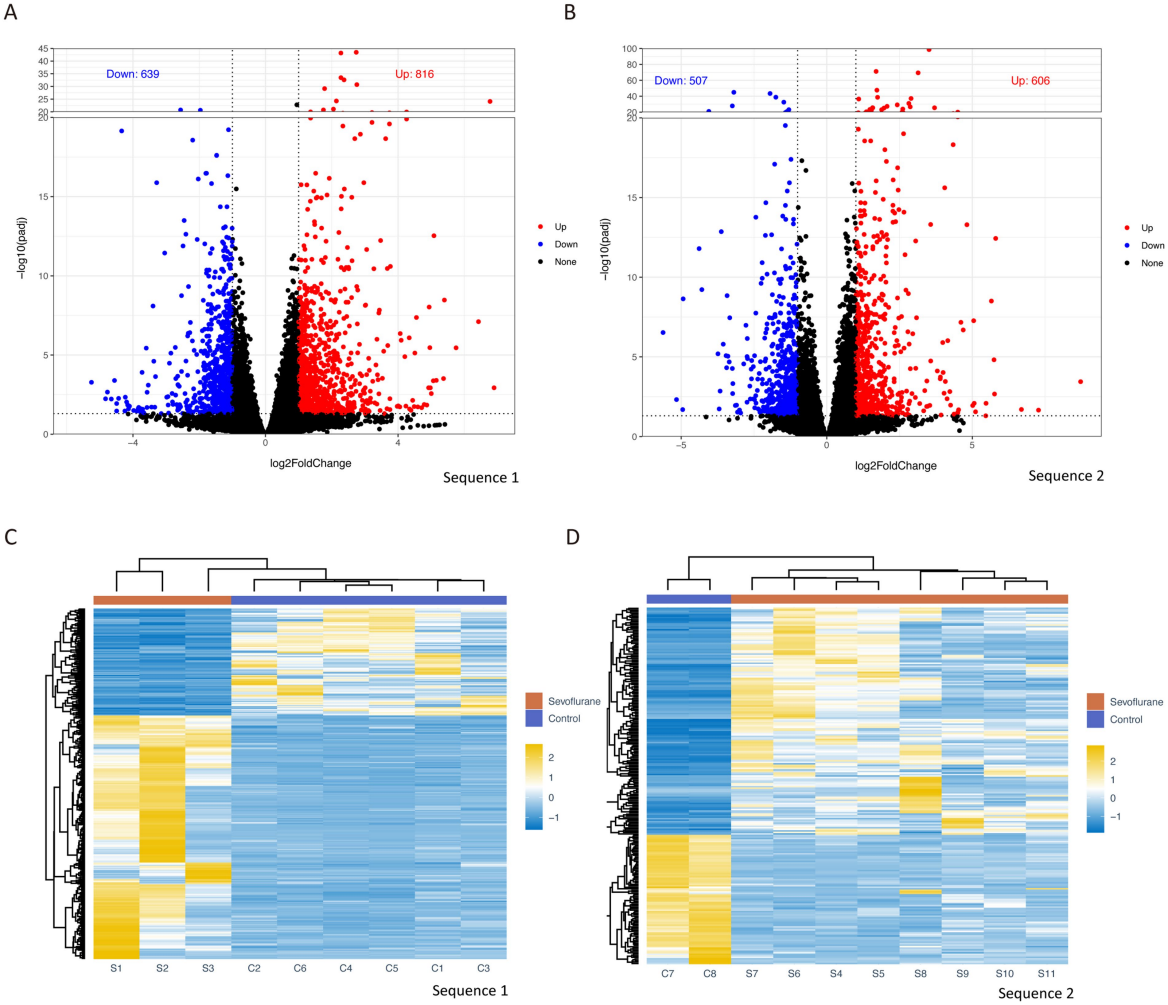
Differential gene analysis volcano plots of differential gene analysis for group 1 sequencing **(A)** and group 2 sequencing **(B)**, with |log2FC| ≥ 1 and P.adjust ≥0.05 as thresholds, log2FC on x-axis and −log10 (P.adjust) on y-axis; heat map based on differential gene expression levels for group 1 **(C)** and group 2 **(D)**, with rows representing different genes and columns representing different samples. Log2FC (log2 fold change).

Differential gene analysis was performed by DEPseq2 for the two mRNA sequencing results of the samples, and differential genes were defined by the threshold of |log2FC| greater than or equal to 1 and a corrected P.adjust less than or equal to 0.05. A total of 816 significantly up-regulated genes and 639 significantly down-regulated genes (Figure 2A) were obtained in the first sequencing group (S1–S3) compared with a control group (C1–C3), and 606 up-regulated genes and 507 down-regulated genes (Figure 2B) were obtained in the second sequencing group (S4–S11) compared with a control group (C4–C8). A heat map of the expression levels of the differentially sequenced genes in the two groups shows a clear distinction between the control group and the sevoflurane group (Figures 2C,D).

### 3.2 Expression analysis of differentially expressed fatty acid metabolism-related genes

**Figure 3:**
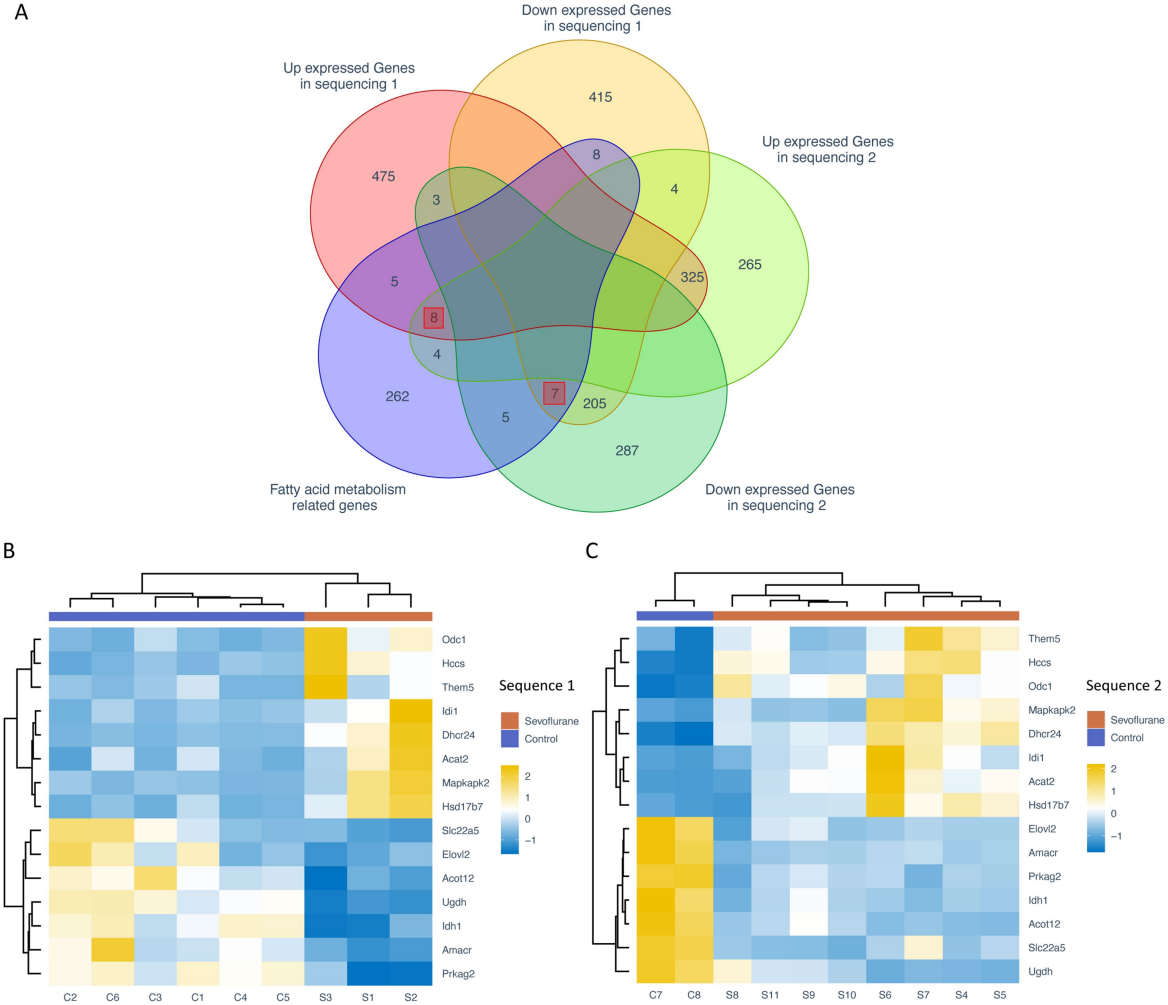
Expression analysis of fatty acid metabolism-related genes. **(A)** Venn diagram of differential genes and intersection of fatty acid metabolism-related genes from two sequencing groups; heat map of fatty acid metabolism-related differential genes from the sevoflurane group **(B)** and the control group **(C)**. Rows represent different fatty acid-associated differential expressed genes and columns represent different samples. The orange annotated bars represent sevoflurane group and the purple annotated bars represent the control group.

To reduce the experimental error, the intersection of the different genes obtained from the two sequencing groups was taken to obtain the co-existing different genes. To detect the expression of fatty acid metabolism-related differential genes, we obtained the list of fatty acid metabolism-related genes based on KEGG, Reactome and Hallmark databases, and further intersected with the list of intersecting differential genes to obtain a total of 8 fatty acid-related differential genes with significantly high expression, such as Acat2, Dhcr24, Hccs, Hsd17b7, Idi1, Odc1, Mapkap1, and Mapkap2. Idi1, Odc1, Mapkapk2 and Them5, and 7 fatty acid-related different genes with significantly low expression, such as Idh1, Slc22a5, Ugdh, Acot12, Amacr, Elovl2 and Prkag2 (Figure 3A). A heat map of the expression of these 15 fatty acid metabolism-related different genes showed that their expression levels were significantly differential expressed between the control and sevoflurane group (Figures 3B,C).

### 3.3 GO and KEGG enrichment analysis of differential genes related to fatty acid metabolism

**Figure 4:**
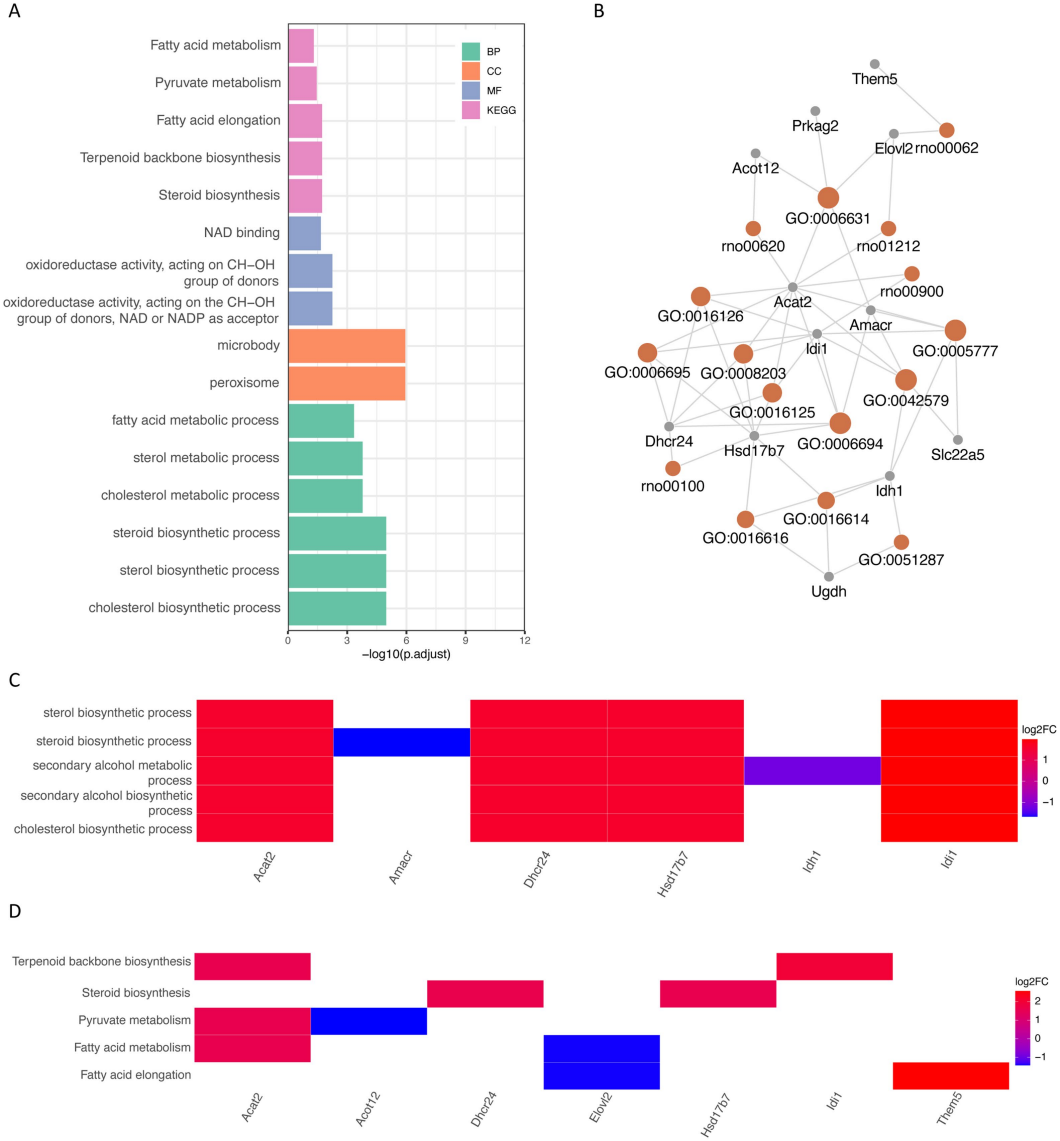
GO and KEGG database ORA analysis **(A)** partial enrichment result histogram of GO and KEGG ORA analysis, the x-axis is −log10 (P.adjust), the y-axis is gene set name, the color represents different gene set types; **(B)** partial gene–gene set association mesh, orange represents gene sets, gray represents differential genes; **(C)** partial GO enrichment results combined with corresponding differential gene logFC heat map; **(D)** KEGG enrichment results combined with corresponding differential gene logFC heat map. GO (Gene Ontology); BP (biological process); CC (cellular component); MF (molecular function); KEGG (Kyoto Encyclopedia of Genes and Genomes); ORA (over representation analysis); Log2FC (log2 fold change).

By using the ClusterProfiler package, we enriched the list of intersecting differential genes based on GO and KEGG databases, and a total of 40 GO genes were significantly enriched, such as: cholesterol biosynthetic process, sterol biosynthetic process and steroid biosynthetic process. Biosynthetic process, etc. In addition, five KEGG pathways were significantly enriched, such as Steroid biosynthesis, Terpenoid backbone biosynthesis and Fatty acid elongation (Figure 4A; Tables 1, 3). Network mapping of the significantly enriched gene set showed that differential expressed genes such as Acat2, Amacr and Dhcr24 are involved in multiple biological pathways simultaneously (Figure 4B). We further combined the different gene log2FC with the enrichment results to show the association between the gene set and the corresponding differential expressed genes in the form of a heat map (Figures 4C,D). log2FC greater than 0 represents up-regulation of gene expression compared to the control group, while less than 0 represents down-regulation.

**Table 3:** KEGG ORA partial enrichment results.

Description	*p* value	p.adjust	*q* value
Steroid biosynthesis	0.00048869	0.018633617	0.014892364
Terpenoid backbone biosynthesis	0.000767892	0.018633617	0.014892364
Fatty acid elongation	0.001035201	0.018633617	0.014892364
Pyruvate metabolism	0.002595976	0.035045672	0.028009212
Fatty acid metabolism	0.00452394	0.048858553	0.039048746

KEGG, Kyoto Encyclopedia of Genes and Genomes; ORA, over-representation analysis.

### 3.4 GSEA and GSVA enrichment analysis

**Figure 5:**
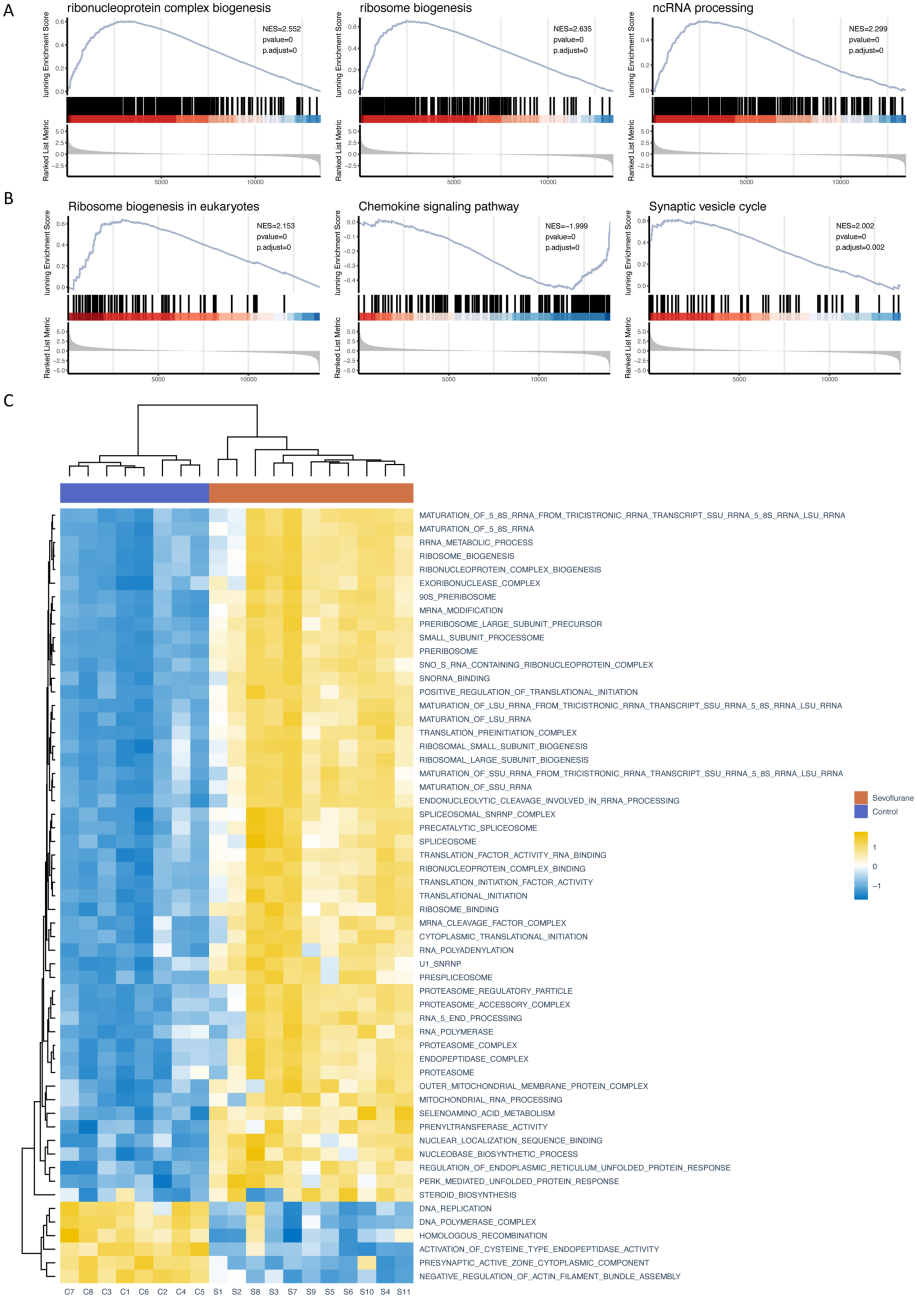
GSEA and GSVA **(A)** partial enrichment score (ES) diagram of GO GSEA (based on the top 3 gene sets sorted by P.adjust); **(B)** partial ES diagram of KEGG GSEA (based on the top 3 gene sets sorted by P.adjust); **(C)** heat map of GSVA, behavior of different gene sets and pathways of GO/KEGG database, listed as different samples. GO (Gene Ontology); KEGG (Kyoto Encyclopedia of Genes and Genomes); GSEA (gene set enrichment analysis); GSVA (gene set variation analysis).

Traditional Over Representation Analysis (ORA) only considers different genes, which might be missed for genes with small variation but large effects. Therefore, we further performed GSEA by the ClusterProfiler package and obtained 126 significantly up-regulated GO gene sets (e.g., cholesterol biosynthetic process and cholesterol metabolic process), 11 KEGG pathways (e.g., Circadian entrainment, etc.), 80 significantly down-regulated GO gene sets (e.g., negative regulation of canonical Wnt signaling pathway, regulation of canonical Wnt signaling pathway and Wnt signaling pathway, etc.) and 12 KEGG pathways (e.g., Chemokine signaling pathways, Cytokine-cytokine receptor interaction and NOD-like receptor signaling pathway, etc.) (Tables 4, 5), with P.adjust less than or equal to 0.05 as the threshold. We then ranked the genes based on P.adjust and visualized the top 3 gene sets (Figures 5A,B). In addition, we performed GSVA based on the GSVA package, and analyzed the expression level of each gene set by limma package, and obtained a total of 130 significantly up-regulated GO gene sets and 5 KEGG pathways and 62 significantly down-regulated GO gene sets and 2 KEGG pathways with a threshold of logFC absolute value greater than or equal to 0.5 and P.adjust less than or equal to 0.05. We then obtained 130 significantly up-regulated GO gene sets and 5 KEGG pathways and 62 significantly down-regulated GO gene sets and 2 KEGG pathways based on the threshold of 0.05. We then ranked the top 50 different GO gene sets and all different KEGG pathways based on P. adjust and drew a heat map (Figure 5C). The results of several gene sets obtained by different enrichment analysis methods are consistent, such as: ribosome biogenesis, ribonucleoprotein complex biogenesis and ribosome binding.

**Table 4:** GO GSEA partial enrichment results.

Description	Ontology	NES	*p* value	p.adjust
Cholesterol biosynthetic process	BP	2.0536663	6.58E-06	0.00135068
Negative regulation of canonical Wnt signaling pathway	BP	−1.6858465	0.00028139	0.01862825
Regulation of canonical Wnt signaling pathway	BP	−1.5629753	0.0004913	0.02578336
Wnt signaling pathway	BP	−1.4119254	0.00061745	0.02976631
Cell–cell signaling by wnt	BP	−1.4125734	0.00070301	0.03230913
Cholesterol metabolic process	BP	1.70620758	0.00078489	0.03463896

GO, gene ontology; GSEA, gene set enrichment analysis; BP, biological process.

**Table 5:** KEGG GSEA partial enrichment results.

Description	NES	*p* value	p.adjust
Chemokine signaling pathways	−1.9988821	0.00000031	0.00005200
Cytokine-cytokine receptor interaction	−1.6837167	0.00007040	0.00387406
NOD-like receptor signaling pathway	−1.5977521	0.00172893	0.02716892
Circadian entrainment	1.69433341	0.00217536	0.03263046

KEGG, Kyoto Encyclopedia of Genes and Genomes; GSEA, gene set enrichment analysis.

### 3.5 Analysis of differential gene networks related to fatty acid metabolism

**Figure 6:**
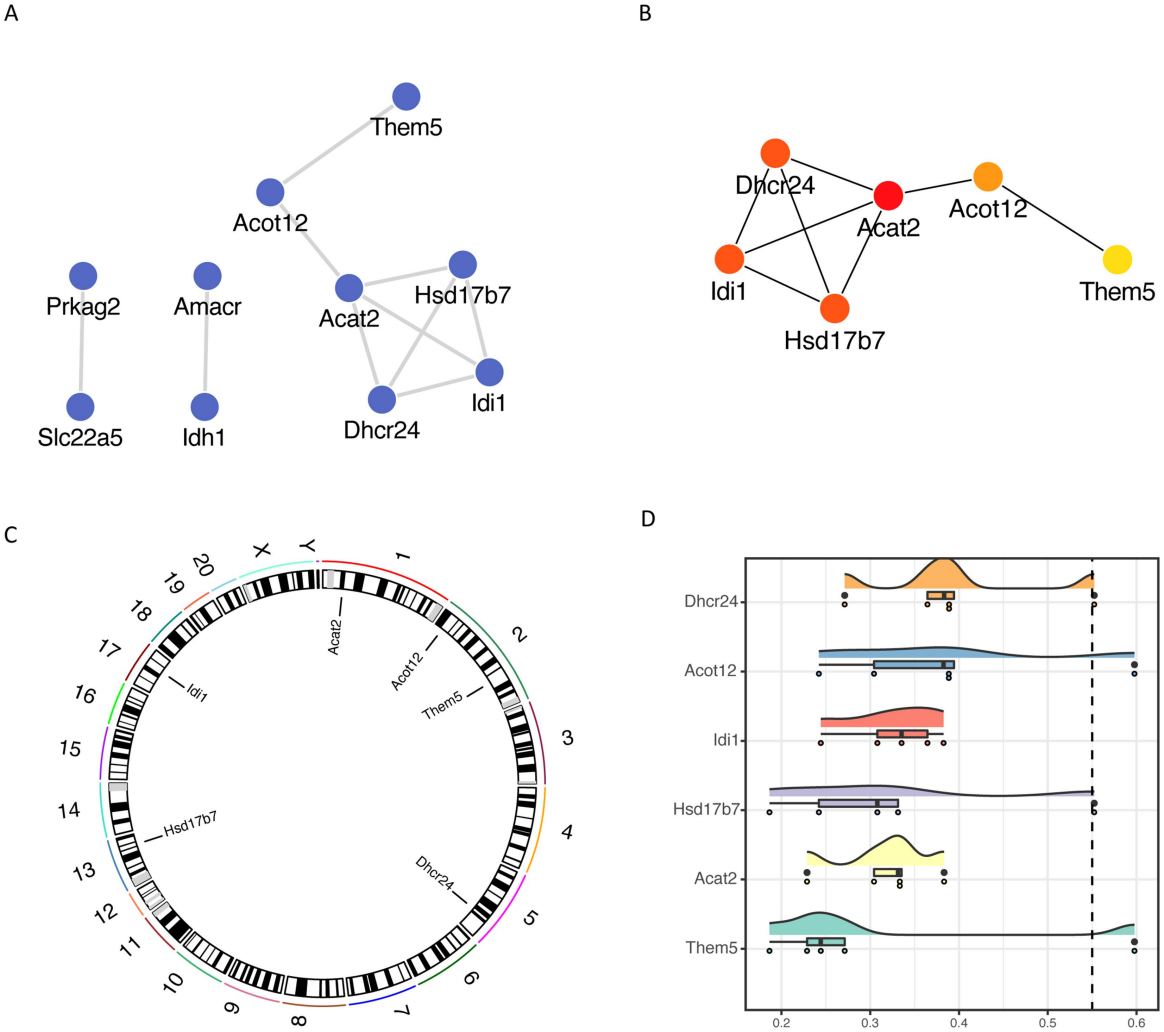
Fatty acid metabolism-associated differential gene network analysis **(A)** protein interaction network of fatty acid metabolism-associated differential genes based on STRING database; **(B)** key genes based on cytoHubba plug-in MCC algorithm; **(C)** chromosomal localization of key genes; **(D)** cloud and rain map of key gene Friends analysis.

We then constructed a protein interaction network based on the STRING database for fatty acid metabolism-related differential genes and visualized them by Cytoscape (Figure 6A), and used the CytoHubba plug-in MCC algorithm to obtain the most critical six different genes as key genes, such as Dhcr24, Acat2, Hsd17b7, Idi1, Acot12 and Them5 (Figure 6B). Acot12 and Them5 (Figure 6B), and visualize the localization of each key gene on the chromosome by RCircos package (Figure 6C).

To compare the importance of different key genes in the pathway, we performed Friends analysis based on the GOSemSim package, suggesting that Dhcr24 might play a more critical role in the pathway (Figure 6D).

### 3.6 Analysis of mRNA-miRNA-lncRNA networks of differential genes related to fatty acid metabolism

**Figure 7:**
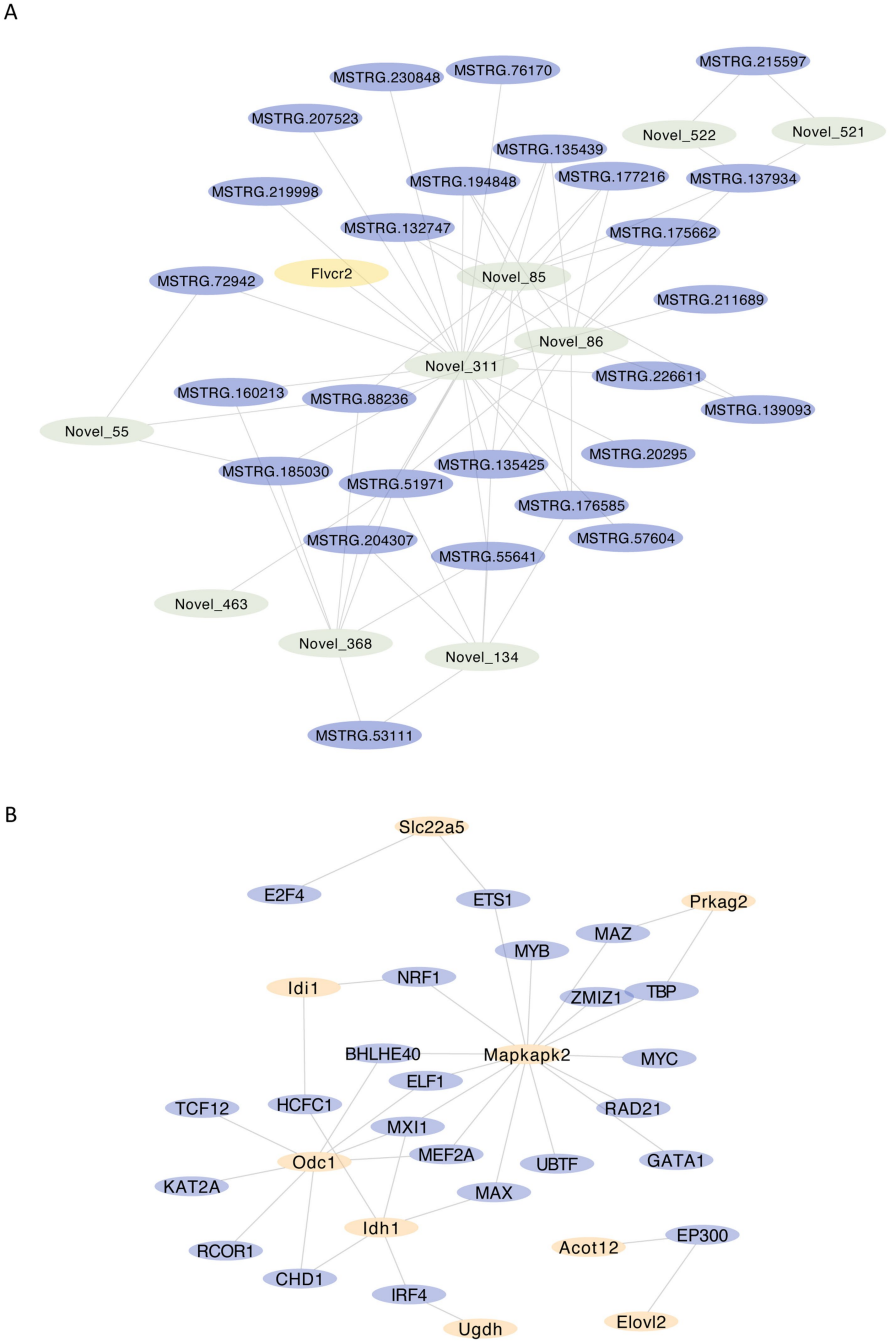
Analysis of the regulatory network of fatty acid metabolism-related differential genes **(A)** mRNA-miRNA-lncRNA network of fatty acid metabolism-related differential genes, visualizing nodes with no less than 110 neighboring nodes within 2 nodes interval, yellow represents mRNA, purple represents lncRNA, green represents miRNA; **(B)** regulatory network of key gene transcription factors, yellow represents key genes, purple represents transcription factors.

In order to explore the regulatory relationship of differential expressed gene network, we further constructed mRNA-miRNA-lncRNA networks for differential genes related to fatty acid metabolism and visualized them by Cytoscape. Novel_311, Novel_85 and Novel_86 in miRNA, MSTRG.51971, MSTRG.177216 and MSTRG.139093 in lncRNA, and Flvcr2 in mRNA play important roles in the regulatory network (Figure 7A).

### 3.7 Analysis of key gene transcription factor networks

Since transcription factors play an important role in gene regulation, we constructed a transcription factor network based on the ENCODE database by NetworkAnalyst for the six key genes (Figure 7B), and the results suggested that transcription factors such as MEFZA, MX11 and ELF1 might play more critical roles in regulation.

### 3.8 Small molecule drug analysis of key genes

**Figure 8:**
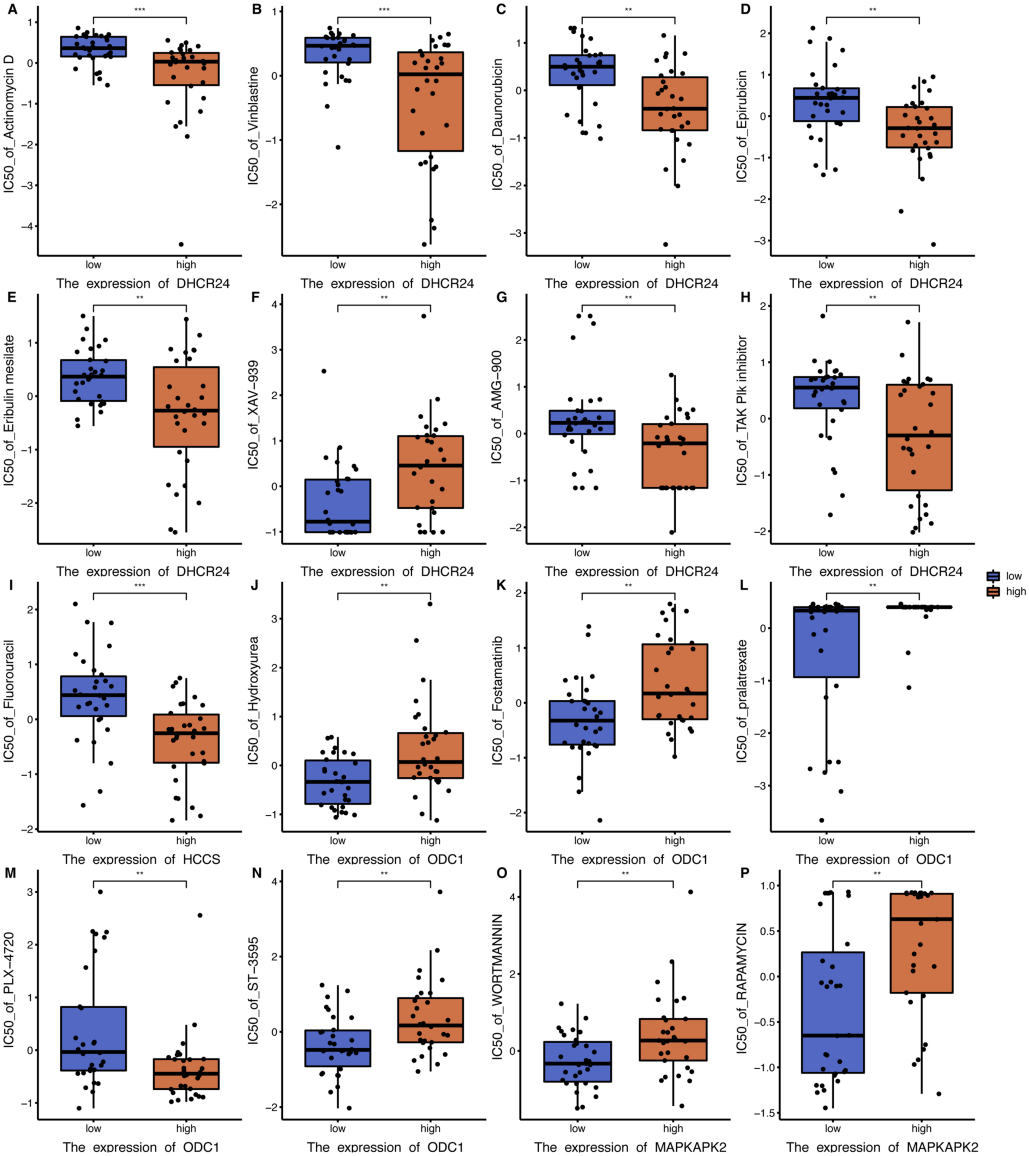
Boxplot of small molecule drug analysis of key genes. **(A)** IC50 boxplots of different DHCR24 expression levels versus actinomycin D resistance. **(B)** IC50 boxplots of different DHCR24 expression levels versus vinblastine resistance. **(C)** IC50 boxplots of different DHCR24 expression levels versus daunorubicin resistance. **(D)** IC50 boxplots of different DHCR24 expression levels versus epirubicin resistance. **(E)** IC50 boxplots of different DHCR24 expression levels versus Eribulin mesylate resistance. **(F)** IC50 boxplots of different DHCR24 expression levels versus XAV-939 resistance. **(G)** IC50 boxplots of different DHCR24 expression levels versus AMG-900 resistance. **(H)** IC50 boxplots of different DHCR24 expression levels versus TAK PIk inhibitor resistance. **(I)** IC50 boxplots of different HCCS expression levels versus fluorouracil resistance. **(J)** IC50 boxplots of different ODC1 expression levels versus hydroxyurea resistance. **(K)** IC50 boxplots of different ODC1 expression levels versus fostamatinib resistance. **(L)** IC50 boxplots of different ODC1 expression levels versus pralatrexate resistance. **(M)** IC50 boxplots of different ODC1 expression levels versus PLX-4720 resistance. **(N)** IC50 boxplots of different ODC1 expression levels versus ST-3595 resistance. **(O)** IC50 boxplots of different MAPKAPK2 expression levels versus wortmannin resistance. **(P)** IC50 boxplots of different MAPKAPK2 expression levels versus rapamycin resistance. * *p* < 0.05; ** *p* < 0.01; *** *p* < 0.001. IC50, half-maximal inhibitory concentration. ODCI, ornithine decarboxylase 1. MAPKAPK2, Mitogen-activated protein kinase-activated protein kinase 2.

To explore the relationship between key genes and small molecule drug sensitivity, RNA expression data (RNA: Rna-Seq) and drug data (Compound activity: DTP NCI-60) from CellMiner database were used, with IC50 values representing drug resistance level. The correlation between key gene expression data and drug small molecule IC50 was examined by Pearson correlation analysis, and key gene-drug small molecule combinations was further screened by the threshold of *p* < 0.01. The Wilcoxon rank sum test was used to investigate whether IC50 values of small molecule drugs were significant differences between the high-expression and low-expression groups of key genes. Defined by *p* < 0.01, there were significant differences in the IC50 of 43 key gene-drug small molecule combinations. For example, high expression of Dhcr24 reduced significantly the resistance to actinomycin D, vinblastine, Danobicin, epirubicin, Eribulin mesylate, AMG-900 and AK Plk inhibitors, while increased significantly the resistance to XAV-939. High expression of HCCS reduced significantly the resistance to Fluorouracil (Figure 8).

### 3.9 Immuno-infiltration analysis

**Figure 9:**
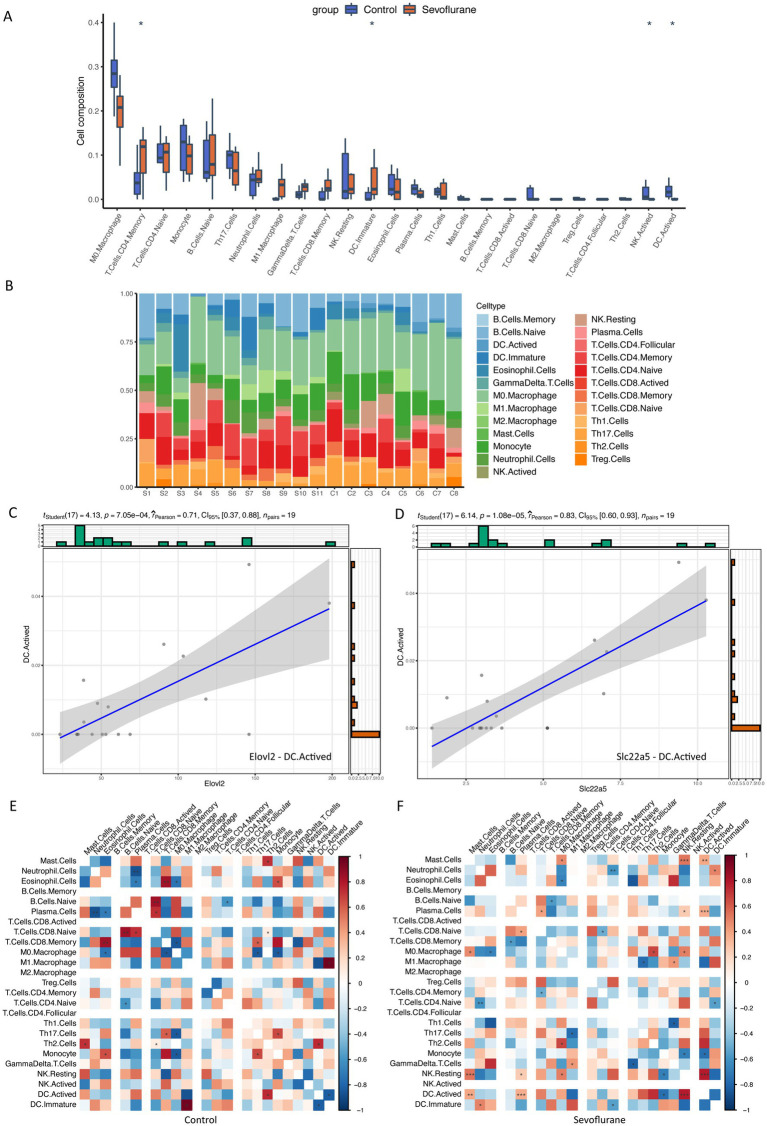
Immune infiltration analysis. **(A)** Box plot of the percentage of each immune cell, purple represents control group, orange represents sevoflurane group; **(B)** Stacked bar graph of immune cell composition of each sample, different colors represent different immune cells; **(C)** Scatter plot of the correlation between Elovl2 gene and active dendritic cells (DC Actived); **(D)** Scatter plot of the correlation between Slc22a5 gene and active dendritic cells (DC Actived); **(E)** Heat map of the correlation between 25 immune cells in control group; **(F)** Heat map of the correlation between 25 immune cells in sevoflurane group cells (DC Actived) correlation scatter plot; **(E)** Correlation heat map of 25 immune cells within control group; **(F)** Correlation heat map of 25 immune cells within the sevoflurane group. * Represents *p* < 0.05, ** represents *p* < 0.01, *** represents *p* < 0.001.

The immune infiltration analysis of each sample was performed by CIBERSORT algorithm based on the marker genes of 25 immune cells obtained from the literature and visualized using box line plots and stacked histograms (Figures 9A,B), which showed that CD4 memory T cells (T Cells CD4 Memory, *p* = 0.018), immature dendritic cells (DC Immature, *p* = 0.031), and active NK cells (NK Actived, *p* = 0.013) were significantly different from active dendritic cells (DC Actived, *p* = 0.018) in the control and sevoflurane group samples. We then examined the correlation between key genes and the level of immune cell infiltration, where a significant positive correlation was found between Elovl2-active dendritic cells (DC Actived, *p* = 0.00071, r2 = 0.71) (Figure 9C) and Slc22a5 – active dendritic cells (DC Actived, *p* = 0.000011, r2 = 0.83) (Figure 9C). A significant positive correlation was found between the two sample groups (Figure 9D). In addition, the correlation between immune cells in the two sample groups showed that some immune cell correlations were altered and the immune microenvironment differed between the two groups (Figures 9E,F), e.g., Neutrophil Cells were negatively correlated with plasma cells in the control group and positively correlated in the sevoflurane group; initial B cells (B Cells Naive) were negatively correlated with CD4 initial T cells (T Cells CD4 Naive) in control group and positively correlated in the sevoflurane group; Th2 cells (Th2 Cells) were positively correlated with CD8 initial B cells (T Cells CD8 Naive) in the control group and negatively correlated in the sevoflurane group. Macrophage (M0 Macrophage) and CD8 memory T cells (T Cells CD8 Memory) were negatively correlated in the control group and positively correlated in the sevoflurane group; Th17 cells (Th17 Cells) and M1 macrophages (M1 Macrophage) were positively correlated in the control group and negatively correlated in the sevoflurane group. Immature dendritic cells (DC Immature) were positively correlated with CD4 initial T cells (T Cells CD4 Naive) in the control group and negatively correlated in the sevoflurane group; monocytes were positively correlated with Th17 cells (Th17 Cells) in the control group and negatively correlated in the sevoflurane group.

### 3.10 RT PCR analysis of liver

**Figure 10:**
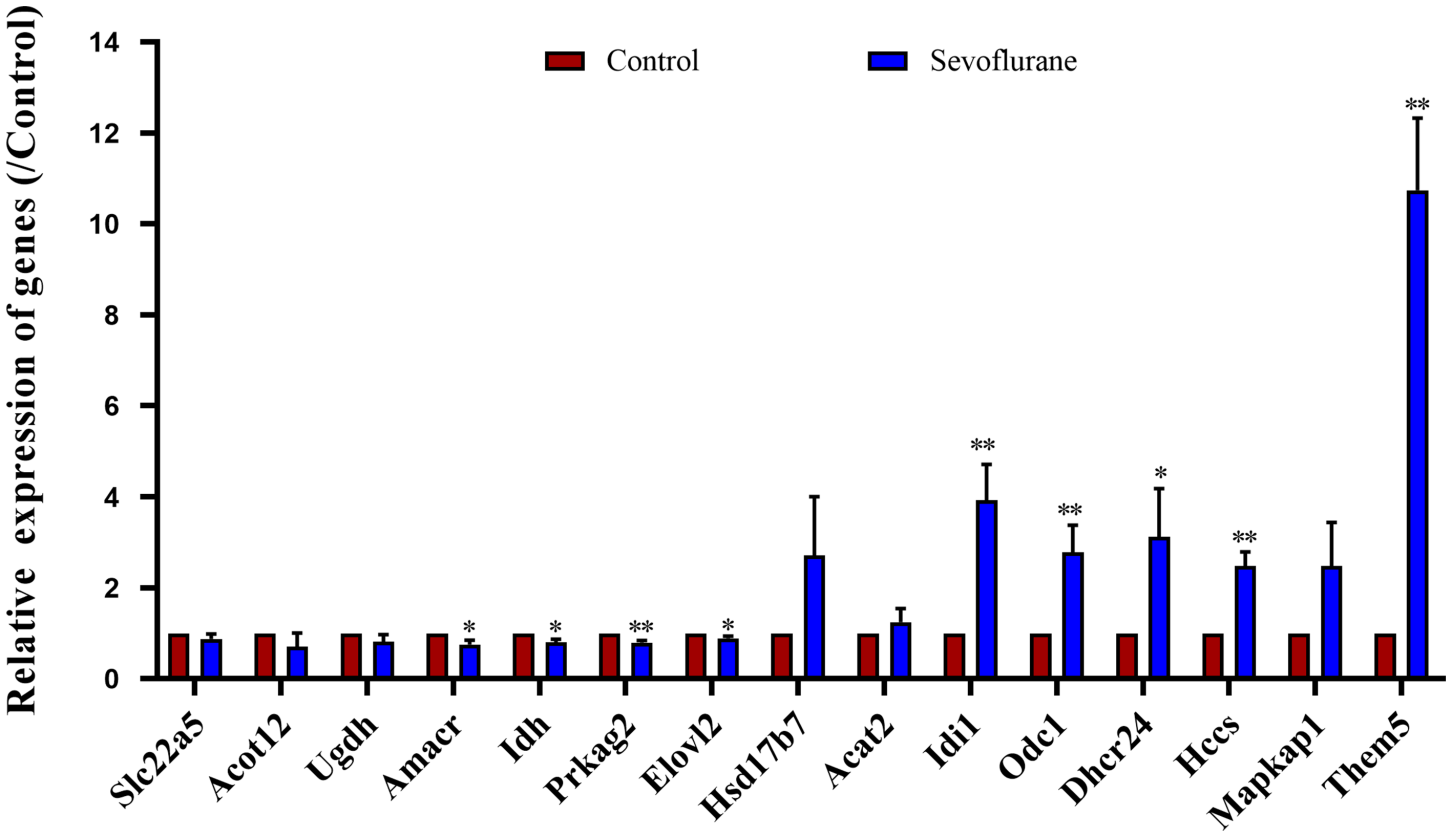
RT-qPCR analysis showed that compared with the control group, the expression of Slc22a5, Acot12, Udgh, Amcar, Idh, Prkag2, Elovl2 genes was relatively reduced and the expression of Dhcr24, Idi 1, Odc 1, Hsd17b7, Mapkap1, HCCS genes was relatively increased in the sevoflurane group. * Represents *p* < 0.05.

Detailed are shown in Figure 10. To further validate the experimental results, we conducted further validation on 15 differentially expressed genes. The results showed that Elovl2 was significantly down regulated in the sevoflurane group (*p* = 0.002), Slc22A5, Acot12, Udgh, Amcar, Idh, Prkag2 were also significantly down regulated, Dhcr24 was significantly up-regulated in the sevoflurane group (*p* = 0.005), Idi 1, Odc 1, Hsd17b7, Mapkap1, HCCS were also significantly up-regulated, which is consistent with the previous experimental results.

### 3.11 Identified by immunohistofluorescence staining

**Figure 11:**
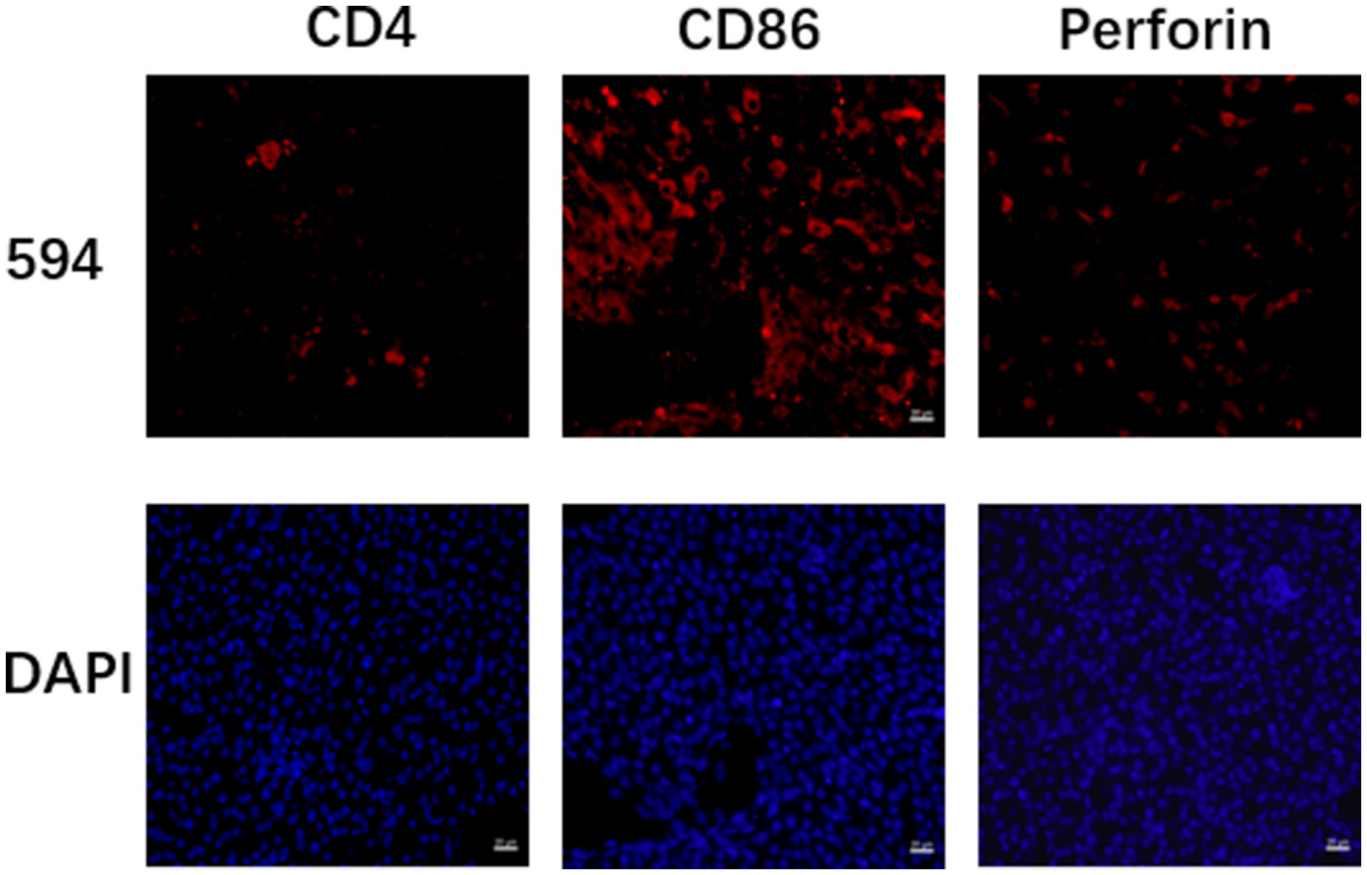
Fluorescence staining results of liver CD4, CD86 and perforin after sevoflurane exposure. Fluorescence staining results of CD4, CD86 and perforin in neonatal 7-day-old rats after exposure to 3.4% sevoflurane for 6 h. The red fluorescent labels are 594-positive cells and the blue fluorescent labels are DAPI-positive cells as shown in the figure. In the figure, scale bars = 20 μm.

Detailed are shown in Figure 11.

## 4 Discussion

This study utilized a validated bioinformatics approach to investigate the impact of metabolic genes associated with liver tissue on brain energy supply following sevoflurane exposure. Some diagnostic biomarkers were also identified. Compared to normal liver tissue, the expression of genes related to fatty acids was significantly altered. These genes were involved in cholesterol, sterol, and steroid biosynthesis. Several biological pathways, such as bile acid signaling, ketone body catabolism, liver development, fatty acid biosynthesis, and sphingolipid biosynthesis, were affected by different genes, including Acat2, Amacr, and Dhcr24. Ketone bodies and fatty acids, which are products related to fatty acid metabolism, are significant sources of energy for brain metabolism (McKenna et al., 2015; Delp et al., 2018). They can be directly taken up and used by the brain. Supplementation with ketone bodies and fatty acids may reduce neonatal hypoxia and ischemia, pediatric traumatic brain injury, and brain damage secondary to prematurity (McKenna et al., 2015; Demers-Marcil and Coles, 2022). Under physiological conditions, hypoglycemia stimulates the secretion of glucagon, which causes the mobilization of fat to produce fatty acids and glycerol (Thorens, 2024). These can be oxidized directly or metabolized by the liver into ketone bodies that are transported outside the liver as an energy substrates (Delezie et al., 2012), which are crucial for providing energy for all cellular processes required for brain development and function. This includes ATP formation, synaptogenesis, neurotransmitter synthesis, release and uptake, maintenance of ion gradients and redox states, and myelination (Etchegaray and Mostoslavsky, 2016; Knobloch, 2017; Ryan et al., 2019). 5 pivotal genes, Dhcr24, Acat2, Hsd17b7, Idi1, Acot12, and Them5, were screened to compare the importance of different key genes in the pathway. It appears that Dhcr24 may play a more critical role in the pathway.

DHCR24 is an important key synthase for cholesterol synthesis in the brain (Ma et al., 2022). Mutations in the DHCR24 gene inhibit its enzyme activity, leading to cholesterol deficiency and accumulation of delipid lipids in the brain (Bai et al., 2022). DHCR24 has been found to be associated with brain and peripheral health properties. Additionally, DHCR24 has a novel role in the metabolism of triglyceride-rich lipoproteins, possibly involving omega-3 fatty acids, particularly DHA, which could affect peripheral metabolism and brain health (Sliz et al., 2021; Mai et al., 2022). However, there is no data to determine the effects of sevoflurane on hepatic cholesterol metabolism. Downregulation in brain regions associated with Alzheimer’s disease has been reported. Seladin-1, a key enzyme in the cholesterol pathway, has been shown to have a neuroprotective effect (Kelicen-Ugur et al., 2019). Additionally, Seladin-1 has been shown to regulate lipid raft formation. A recent study showed that Dhcr24 affects energy transfer between neurons and astrocytes by promoting cholesterol metabolic differentiation. Overexpression of DHCR24 prevented the overactivation of Ras/MEK/ERK signaling by increasing cellular cholesterol content, thereby decreasing tau hyperphosphorylation in astrocytes (Mai et al., 2022). Research has shown that developing cortical neurons rely on endogenous cholesterol synthesis and utilize apolipoprotein complex cholesterol and sterol precursors from their surrounding environment. Both developing neurons and astrocytes release cholesterol into their local environment (Genaro-Mattos et al., 2019). Additionally, it was discovered that Dhcr24 is highly expressed mainly in neonatal murine livers exposed to sevoflurane. Therefore, as Dhcr24 plays a vital role in abnormal cholesterol metabolism, it could serve as a diagnostic biomarker.

In the present study, we observed significant changes in the expression of genes related to fatty acid metabolism in rat liver after sevoflurane exposure. This finding suggests that sevoflurane may indirectly affect brain development by affecting energy metabolism in the liver. Specifically, the rat model used in the experiment showed that some key genes played a crucial role in fatty acid metabolism, and the pattern of changes in these genes was consistent with human physiological characteristics, especially in the growth and development of children with high energy requirements to support their rapid growth and brain development (Chen et al., 2022). This study reveals a potential mechanism of sevoflurane influencing fatty acid metabolism: by changing the activity level of key enzymes or regulators involved in fatty acid synthesis, decomposition and transport in the liver, the energy balance of the whole body is affected, which provides a new perspective for understanding how anesthetics act on non-target organs. In addition, it is worth noting that the above changes in specific molecular markers in fatty acid metabolic pathways not only help to explore the biological basis behind sevoflurane-induced cognitive dysfunction or other neurodevelopmental abnormalities, but also open the way for the development of new diagnostic tools.

In this study, CIBERSORT algorithm was used to analyze the immune infiltration of different samples. It was shown that there were significant differences in the infiltration level of various immune cells between two groups. Initial B cells were negatively correlated with CD4 initial T cells in the control group and positively correlated in sevoflurane group. In the control group, Th2 cells showed a positive correlation with CD8 initial B cells, while in sevoflurane group, they showed a negative correlation. Similarly, Th17 cells and M1 macrophages were positively correlated in the control group but negatively correlated in sevoflurane group. Immature dendritic cells were positively correlated with CD4 initial T cells in the control group and negatively correlated in sevoflurane group. In the control group, monocytes showed a positive correlation with Th17 cells, while in sevoflurane group, they showed a negative correlation. This suggests that exposure to sevoflurane causes significant changes in T lymphocytes and NK cells, which in turn affects immune function and may be associated with the occurrence of neurodevelopmental abnormalities. Moreover, two important genes related to fatty acid metabolism, Elovl2 and Slc22a5, were highly consistent with the number of activated dendritic cells, indicating that the fatty acid metabolic pathway may be a key link in regulating immune activity.

The 15 differentially expressed genes closely related to fatty acid metabolism (including but not limited to Acat2, Dhcr24, etc.) screened from this study are expected to become new indicators for assessing the nervous system health of patients in the future. Regular monitoring of the changes in the expression levels of these genes is helpful for early identification of those who are at higher risk of developing neurodevelopmental disorders and timely intervention (Elena et al., 2003). Given the importance of fatty acid metabolism in maintaining normal immune function, it is particularly important to develop targeted therapies for this process. For example, the potential harm caused by narcotic drugs can be mitigated by optimizing the energy supply of the brain by supplementing specific types of fatty acids or by using drugs that enhance the production of endogenous fatty acids (Etchegaray and Mostoslavsky, 2016). In addition, improving the overall nutritional status is also one of the effective means to improve the treatment effect. Finally, based on these findings, future research should focus more on exploring how to effectively manage the immune response of perioperative patients. This includes, but is not limited to, finding new immunomodulators and improving existing treatment regimens to reduce the incidence of side effects. Fine regulation of immune system function can not only reduce the incidence of complications, but also significantly improve the quality of recovery after surgery, and ultimately achieve the purpose of improving the prognosis.

This study identified several diagnostic biomarkers for hepatic fatty acid metabolism and immune cell infiltration after sevoflurane exposure. However, there were some limitations as following: (1) the data mining nature based on genome sequencing. This means that when we used the relevant genes to create credible predictive features, we did not conduct independent experimental studies to further validate these findings. For example, we did not perform cellular or animal experiments to confirm directly the existence of reliable predictive features. Therefore, our findings and conclusions need to be verified and strengthened by more experimental studies. (2) the data in this study was derived entirely from genome sequencing, which may lead to the problem of insufficient sample size. (3) the study did not use internal validation, such as using cross-validation or Bootstrap, to ensure that the results were not generated by chance. (4) Current animal studies on neurodevelopmental damage caused by sevoflurane have focused on small animals and primates, and the results of whether similar reactions exist in clinical settings and how population factors such as genetics, environment, and lifestyle may influence sevoflurane exposure have not yet been widely verified.

In conclusion, a total of 15 fatty acid metabolism-related differential genes including Acat2, Dhcr24, Hccs, Hsd17b7, Idi1, Odc1, Mapkap1, and Mapkap2, Idh1, Slc22a5, Ugdh, Acot12, Amacr. Elovl2 and Prkag2 might be potential diagnostic and therapeutic biomarkers for neurodevelopmental abnormalities after sevoflurane exposure. Acat2, Amacr and Dhcr24 were involved in multiple biological pathways, and Dhcr24 might play a more critical role in the pathway. These changes in liver lipid metabolism suggested a potential mechanism for neurodevelopmental toxicity of sevoflurane.

## Data Availability

All data generated or analyzed are included in this article and its supplementary files. Detailed expression matrices and analysis pipelines can be obtained from the corresponding author upon reasonable request.
